# Long Noncoding RNAs Coregulated by Annexin A7 and JNK in Hepatocellular Carcinoma Cells Identified by Whole-Genome Expression Profiling

**DOI:** 10.1155/2020/5747923

**Published:** 2020-07-25

**Authors:** Qi Deng, Lianhong Li, Yanling Jin

**Affiliations:** ^1^Department of Pathology, First Affiliated Hospital of Dalian Medical University, No. 222, Zhongshan Road, Dalian, Liaoning, China; ^2^Department of Pathology, Dalian Medical University, China

## Abstract

Knockdown of Annexin A7 (ANXA7) or C-Jun N-terminal kinase (JNK) inhibits the proliferation, migration, invasion, and lymphatic adhesion of hepatocellular carcinoma (HCC) cells, suggesting that ANXA7 and JNK signaling pathways contribute to HCC growth and lymph node metastasis (LNM). While the intervening molecular pathways are largely unknown, emerging evidence suggests that long noncoding RNAs (lncRNAs) participate in ANXA7 and JNK signaling. To identify potential therapeutic targets for HCC, we screened for lncRNAs differentially expressed among Hca-P cells stably expressing shRNA-ANXA7, shRNA-JNK, or control-shRNA. RNA sequencing identified 216 lncRNAs differentially expressed between shRNA-ANXA7 and control-shRNA cells, of which 101 were downregulated and 115 upregulated, as well as 436 lncRNAs differentially expressed between shRNA-JNK and control-shRNA cells, of which 236 were downregulated and 200 upregulated. Fifty-six lncRNAs were differentially expressed under both ANXA7 and JNK knockdown. We selected 4 of these for verification based on putative involvement in cancer regulation according to GO and KEEG analyses of target genes. Knockdown of ANXA7 or JNK suppressed expression of NONMMUT012084.2, NONMMUT024756.2, and ENSMUST00000130486, and enhanced expression of ENSMUST00000197932. These lncRNAs are intriguing candidate targets for mechanistic analysis of HCC progression and therapeutic intervention.

## 1. Introduction

Hepatocellular carcinoma (HCC) is the fifth most frequent cancer globally [1] and the most frequent malignant liver tumor [2]. Both HCC morbidity and mortality continue to increase [3], so novel treatment strategies are urgently required. Previous studies have shown that ANXA7 and JNK are involved in lymphatic metastasis of HCC, suggesting downstream genes as potential therapeutic targets [4, 5].

Annexin A7, also termed ANXA7 or synexin, is ubiquitously expressed in the liver as 47 kDa and 51 kDa isoforms [6]. It is a Ca^2+^/phospholipid-binding protein that facilitates membrane aggregation, fusion, adhesion, and transport. ANXA7 can also activate GTPases to drive Ca^2+^/GTP signal transduction pathways [7]. Numerous studies have suggested that ANXA7 is a promoter of mouse HCC lymphatic metastasis [8–10] as downregulation suppresses the growth, differentiation, proliferation, secretion, invasion, and migration of HCC cells while enhancing apoptosis [6, 11–14].

C-Jun N-terminal kinase (JNK), a member of the mitogen-activated protein kinase (MAPK) family [15], regulates multiple cellular processes including apoptosis by phosphorylating transcription factors such as ATF-2 [16], c-Myc [17], p53 [18], Elk1 [19], and NFAT [20], which in turn regulate a multitude of cellular responses, such as proliferation, differentiation, metastasis, and apoptosis. Previous findings have shown that activation of JNK can promote the proliferation and inhibit the apoptosis of hepatoma cells [5, 21–23], suggesting JNK a promising target for HCC therapy.

Long noncoding RNAs (lncRNAs) are 200-nt or more transcripts that do not encode proteins but regulate coding gene expression, thereby influencing many diverse biological processes including disease pathogenesis [24–27]. Dysregulated expression of HCC-related lncRNAs such as MEG-3 [28], MALAT1 [29], HULC [30], HOTAIR [31], and H19 [32] have been linked to tumorigenesis and metastasis. Compared with normal liver tissue, a large number of lncRNAs were abnormally expressed in HCC. LncRNAs imbalance will lead to chronic hepatitis, excessive liver growth, and oxidative stress response, and ultimately lead to the occurrence of HCC. These species may therefore serve as valuable biomarkers for prognosis and diagnosis [27] as well as potential therapeutic targets.

In the past 10 years, high-throughput RNA sequencing (RNA-seq) has been widely used for lncRNA detection and functional analysis in various diseases (including tumors). Weighted gene coexpression network analysis (WGCNA) is a bioinformatics strategy widely used for finding coexpressed gene modules and exploring the correlation between gene networks and phenotypes of interest [33]. While a few previous studies have examined the associations among ANXA7, JNK, and lncRNAs in HCC, it remains unclear how ANXA7 and JNK regulate specific lncRNAs and the contributions of individual species to HCC. This study screened for lncRNAs differentially expressed in HCC cells under ANXA7 and JNK knockdown to identify potential therapeutic targets and provide clues to molecular mechanisms linking ANXA7 and JNK to HCC progression.

Previous studies have found that the JNK/ANXA7 signaling pathway is expressed in hepatocellular carcinoma cells. Downregulation of JNK and ANXA7 can inhibit the proliferation, migration, and invasion of HCC cells. JNK and ANXA7 may concurrently play a role in lymphatic metastasis of HCC. To further investigate the mechanism, this study explored the simultaneous regulation of lncRNA by JNK and ANXA7 to lay a foundation for future studies.

## 2. Materials and Methods

### 2.1. Cell Lines and Cell Culture

The mouse HCC cell line Hca-P with low lymphatic metastatic potential was established and maintained by the Key Laboratory of Tumor Metastasis in Liaoning Province. Hca-P cells were routinely cultured in 90% RPMI 1640 medium (Invitrogen, USA) supplemented with 1% gentamicin/streptomycin (Beyotime, China) and 10% fetal bovine serum (Gibco, USA) in an incubator (Thermo, USA) under a humidified 5% CO_2_ atmosphere at 37°C.

### 2.2. Transient and Stable Transfection

Expression levels of ANXA7 and JNK were separately downregulated in Hca-P cells by the transfection of targeted shRNA expression vectors. Briefly, the shRNA-ANXA7 expression plasmid 5′-CACCGTCAGAATTGAGTGGGAATTTCAAGAGAATTCCCACTCAATTCTGACTTTTTTG-3′ and the shRNA-JNK expression plasmid 5′-CACCGCAGGCCTAAATACGCTGGATTCAAGAGATCCAGCGTATTTAGGCCTGTTTTTTG-3′ were inserted separately into the pGPU6/GFP/Neo-shRNA expression vector (expressing green fluorescent protein [GFP] SS and the Neo resistance cassette), yielding pGPU6/GFP/Neo-shRNA-ANXA7 (termed shRNA-ANXA7) and pGPU6/GFP/Neo-shRNA-JNK (shRNA-JNK). A nonspecific shRNA pGPU6/GFP/Neo vector was used as a control (control-shRNA) for both targeted vectors. Plasmids were designed by Genepharma Co., Ltd. (Shanghai, China). Hca-P cells were seeded at 5 × 10^5^/well in 24-well plates with serum-free RPMI-1640 medium overnight and then transfected with the indicated vector using Lipofectamine 2000 (Invitrogen, USA) according to the manufacturer's instructions. After 24 and 48 h, the transfection efficiency was examined by observing GFP emission using a fluorescence microscope (Olympus, Japan). Stably transfected cell lines were obtained by growing transfected cells in RPMI 1640 medium supplemented with 10% fetal bovine serum (Gibco) and 400 g/mL G418 (Gibco) for 28 days. A portion of the medium was replaced daily during selection. When cells reached near confluence, they were split into two or three cultures with transfection medium and maintained as described.

### 2.3. Establishment and Sequencing of RNA Libraries

Total RNA was extracted using Trizol reagent (TakaRa, Japan) according to the manufacturer's instructions. Strand-specific libraries were prepared using the TruSeq® Stranded Total RNA Sample Preparation kit (Illumina, USA) following the manufacturer's instructions. Briefly, ribosomal RNA was removed from total RNA using Ribo-Zero rRNA removal beads. Following purification, the mRNA is fragmented into small pieces using divalent cations under 94°C for 8 min. The cleaved RNA fragments are copied into first-strand cDNA using reverse transcriptase and random primers. This is followed by second-strand cDNA synthesis using DNA Polymerase I and RNase H. These cDNA fragments then go through an end repair process, the addition of a single “A” base, and then ligation of the adapters. The products are then purified and enriched with PCR to create the final cDNA library. Purified libraries were quantified by Qubit® 2.0 Fluorometer (Life Technologies, USA) and validated by Agilent 2100 bioanalyzer (Agilent Technologies, USA) to confirm the insert size and calculate the mole concentration. Cluster was generated by cBot with the library diluted to 10 pM and then were sequenced on the Illumina HiSeq (Illumina, USA). The library construction and sequencing was performed at Shanghai Biotechnology Corporation.

### 2.4. Analysis of Differentially Expressed lncRNAs

The ballgown package in R language was used to analyze the expression differences between StringTie assembled and quantified genes (*P* < 0.05). LncRNAs with relative expression|log2fc|≥1, where fc is fold change, and *P* < 0.05 were defined as differentially expressed.

### 2.5. Extraction and Expression Analysis of Target lncRNAs

Stably transfected cells were rinsed twice with ice-cold PBS and subjected to total RNA extraction using Trizol reagent (TakaRa, Japan) according to the manufacturer's instructions. RNA concentration was determined by the absorbance at 260 nm. An 800-ng sample of total RNA from each transfection group was reverse transcribed using the All-in-one First-strand cDNA Synthesis Kit (Transgen, China) and a thermocycle of 42°C for 15 min and 85°C for 5 s. Expression levels of NONMMUT012084.2, NONMMUT024756.2, ENSMUST00000197932, and ENSMUST00000130486 (identified as differentially expressed and potentially involved in HCC) were measured by quantitative reverse transcription-polymerase chain reaction (qRT-PCR) using Power SYBR Green PCR Master Mix (Thermo Fisher, USA). Relative expression was calculated using the comparative *^ΔΔ^*Ct method with GAPDH expression as the internal control. The primer sequences for NONMMUT012084.2, NONMMUT024756.2, ENSMUST00000197932, ENSMUST00000130486, and GAPDH are listed in Table 1.

### 2.6. Prediction and Functional Analysis of LncRNA Target Genes

Most lncRNAs have no known function. However, possible functions may be predicted based on downstream target genes. Long noncoding RNAs regulate downstream target genes via two known modes, cis and trans. The target genes of cis-mode lncRNA regulation are usually located within 1 × 10^5^ bp up- or downstream of the lncRNA locus, while the target genes of trans-mode lncRNA regulation are on different chromosomes and must be predicted by the free energy binding to RNA secondary structure. In this study, BLAST2GO and Kyoto Encyclopedia of Genes and Genomes (KEGG) were used to predict target genes and reveal potential functions of lncRNAs.

### 2.7. Weighted Gene Coexpression Network Analysis

After preprocessing of lncRNA expression data, Pearson's correlation coefficients were calculated between each pair, followed by determination of the adjacency function and cluster analysis to identify gene modules. Weighted coexpression network analysis was then applied to construct a coexpression network.

### 2.8. Statistical Analysis

Statistical analyses were conducted using SPSS software (version 22.0, IBM, USA). All data are shown as mean ± standard deviation (SD). Group means were compared by independent samples *t*-tests. A *P* < 0.05 (two-tailed) was considered statistically significant for all tests.

## 3. Results

### 3.1. Determination of Transfection Efficiency

**Figure 1:**
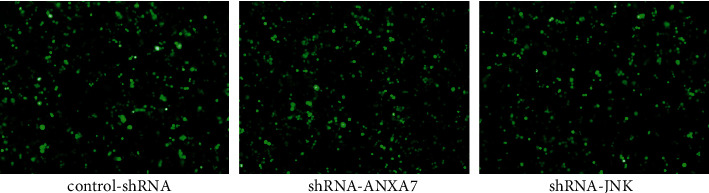
Transfection efficiency of control-shRNA, shRNA-ANXA7, and shRNA-JNK Hca-P cell groups as verified by green fluorescence protein emission from the pGPU6/GFP/Neo shRNA vector at 48 h (magnification, 100x).

The transfection efficiencies of the constructed pGPU6/GFP/Neo-shRNA expression vectors were evaluated by GFP fluorescence emission. Examination under inverted epifluorescence microscopy revealed that around 70% of surviving cells in all three treatment groups (control-shRNA, shRNA-ANXA7, and shRNA-JNK) emitted green fluorescence after 48 h of transfection (Figure 1). Stably transfected cell lines with shRNA-mediated ANXA7 or JNK knockdown were then established by 28 days of continuous G418 selection.

### 3.2. Differentially Expressed lncRNAs under ANXA7 or JNK Knockdown

**Figure 2:**
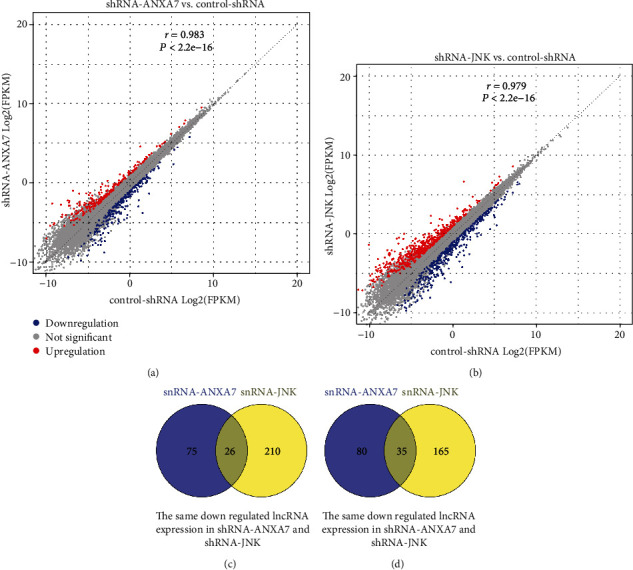
Long noncoding RNAs (lncRNAs) differentially expressed under ANXA7 or JNK knockdown in Hca-P hepatocellular carcinoma cells. (a). Express correlation scatter plot for the shRNA-ANXA7 group vs. control-shRNA group. Red represents upregulated, and blue represents downregulated lncRNAs. (b). Express correlation scatter plot for the shRNA-JNK group vs. control-shRNA group. Red represents upregulated, and blue represents downregulated lncRNAs. (c). Common lncRNAs downregulated by both ANXA7 and JNK knockdown. (d). Common lncRNAs upregulated by both ANXA7 and JNK knockdown.

In total, 216 lncRNAs were differentially expressed between shRNA-ANXA7 and control-shRNA Hca-P cell lines, with 115 upregulated and 101 downregulated, and 436 lncRNAs were differentially expressed between shRNA-JNK and control-shRNA cells, with 200 upregulated and 236 downregulated. Among these, 35 lncRNAs were upregulated by both ANXA7 and JNK knockdown, while 26 were downregulated by both ANXA7 and JNK knockdown (Figures 2(a)–2(d)).

### 3.3. GO Analysis of Differentially Expressed lncRNA Target mRNAs

**Figure 3:**
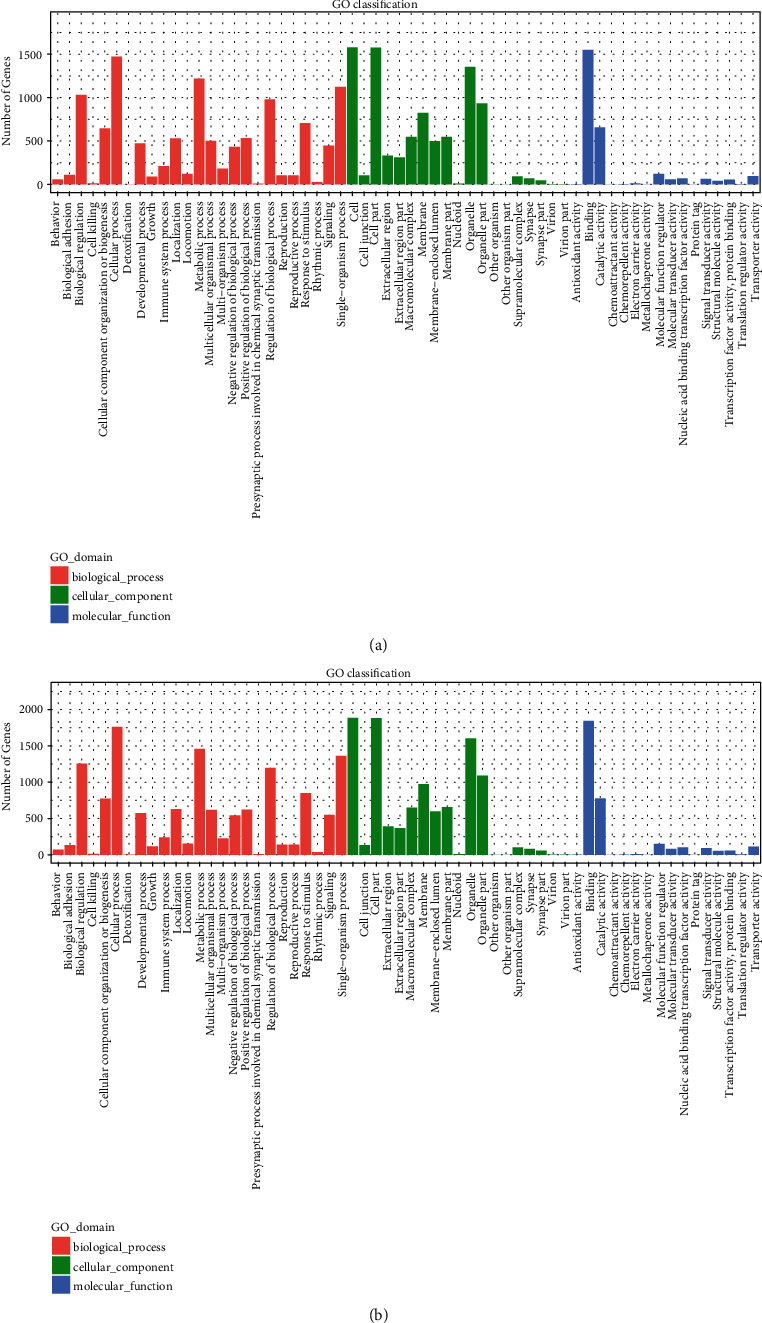
GO functional classification statistics of differentially expressed lncRNA target mRNAs. (a). shRNA-ANXA7 vs. control-shRNA. (b). shRNA-JNK vs. control-shRNA.

The potential biological functions of ANXA7- and JNK-regulated lncRNAs were examined by BLAST2GO classification of putative downstream target mRNAs (Figures 3(a) and 3(b)). Biological processes associated with the greatest number of genes targeted by these differentially expressed lncRNAs included “biological regulation,” “cellular process,” “metabolic process,” “regulation of biological process,” “response to stimulus,” and “single-organism process.” Based on GO prediction, these differentially expressed lncRNAs were associated primarily with “biological membranes and organelles,” where they regulate “binding,” “catalytic activity,” “molecular function,” “molecular transducer activity,” “signal transducer activity,” “transporter activity,” and “nucleic acid binding transcription factor activity” (Figures 3(a) and 3(b)).

### 3.4. Enrichment of Differentially Expressed lncRNAs and Downstream Target Genes in KEGG Pathways

**Figure 4:**
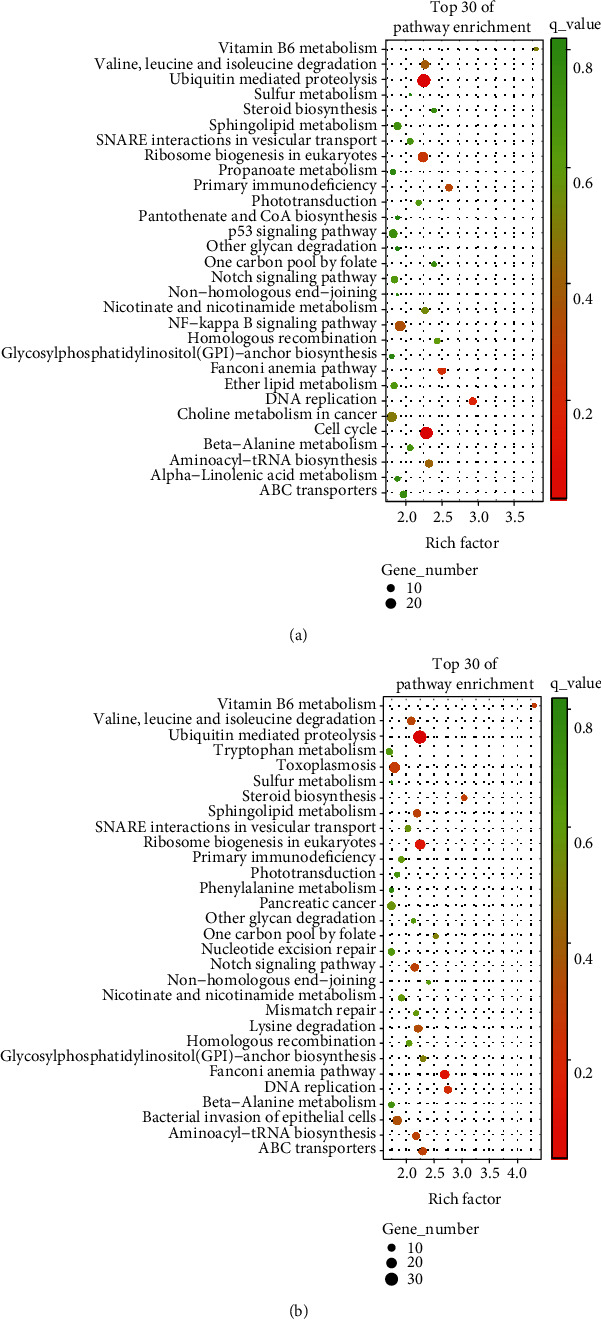
KEEG enrichment scatter plots of differentially expressed lncRNA target genes. (a). Top 30 KEGG pathways for target genes in the shRNA-ANXA7 cell group. (b). Top 30 KEGG pathways for target genes in the shRNA-JNK cell group.

The downstream target genes of lncRNAs can be predicted according to cis- and transregulation modes. In this study, the target genes of transregulation by lncRNAs located on other chromosomes were predicted by the free energy of RNA secondary structure, while the 1 × 10^5^ bp spans upstream and downstream of lncRNAs were assessed for potential target genes of cis-regulation. In turn, the signaling pathways and processes associated with these genes can reveal the general biological functions of the lncRNAs. In the shRNA-ANXA7 gene group, the top 30 KEGG pathways included “ubiquitin mediated proteolysis,” “cell cycle,” and “DNA replication” (Figure 4(a)), while in the shRNA-JNK gene group, the top 30 KEGG pathways included “ubiquitin-mediated proteolysis,” “Fanconi anemia pathway,” and “ribosome biogenesis in eukaryotes” (Figure 4(b)).

### 3.5. Weighted Gene Coexpression Network Analysis of Common Differentially Expressed lncRNAs

**Figure 5:**
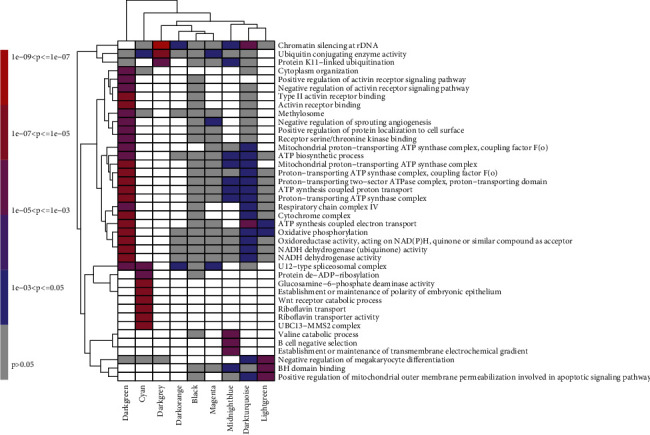
All modules rich factor summation sequence top 40 GO term enrichment heat map.

Target genes in each WGCNA module were mapped according to terms in the GO database, the number of genes in each item was calculated, and then hypergeometric tests were applied to screen for GO items significantly enriched in the module containing genes compared to the whole genomic background (Figure 5).

### 3.6. Knockdown of ANXA7 or JNK Reduced NONMMUT012084.2, NONMMUT024756.2, and ENSMUST00000130486 lncRNA Expression

**Figure 6:**
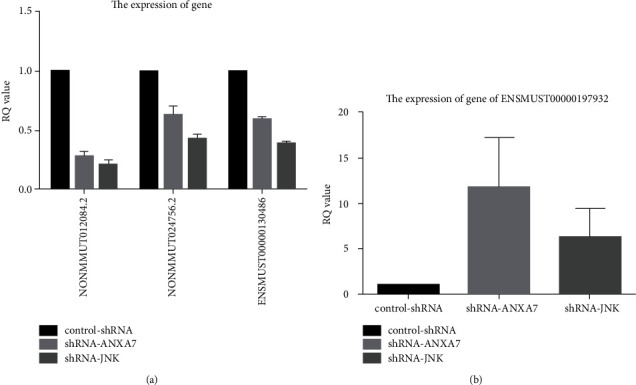
(a) Relative expression levels of the lncRNAs NONMMUT012084.2, NONMMUT024756.2, and ENSMUST00000130486 measured by qRT-PCR following stable transfection of Hca-P cells with control-shRNA, shRNA-ANXA7, or shRNA-JNK (*P* < 0.05). (b) Relative expression level of ENSMUST00000197932 (*P* < 0.05).

Expression levels of the lncRNAs NONMMUT012084.2, NONMMUT024756.2, and ENSMUST00000130486 were significantly reduced by both ANXA7 and JNK knockdown in Hca-P cells as evidenced by qRT-PCR (NONMMUT012084.2: 27.7% ± 4.2% and 20.9% ± 3.4%; NONMMUT024756.2: 62.8% ± 7.3% and 43.0% ± 3.5%; ENSMUST00000130486: 59.4% ± 1.3% and 39.4% ± 0.8%, respectively, of control-shRNA; all *P* < 0.05) (Figure 6(a)).

### 3.7. Knockdown of ANXA7 or JNK Increased ENSMUST00000197932 lncRNA Expression

Expression of the lncRNA ENSMUST00000197932 was significantly enhanced by both ANXA7 and JNK knockdown in Hca-P cells as evidenced by qRT-PCR (1175.4% ± 547.6% and 634.2% ± 313.5%, respectively, of control-shRNA; both *P* < 0.05) (Figure 6(b)).

### 3.8. Predicted Cis-Regulated Target Genes of Differentially Expressed lncRNAs and RNA-Sequencing Results of Cis-Regulated Target Genes

The target genes of cis-regulation by differentially expressed lncRNAs were predicted based on regional sequence analysis (Table 2). The cis-targeted gene of NONMMUT012084.2 is *Rdm1*, the expression of *Rdm1* was significantly reduced by JNK knockdown, and the change was not statistically significant by ANXA7 knockdown in Hca-P cells as evidenced by RNA-sequencing (fold change is 0.667 and 4.157, respectively, of control-shRNA; *P* < 0.01 and *P* > 0.05, respectively). The cis-targeted gene of NONMMUT024756.2 is *Irak4,* the expression of *Irak4* was reduced by JNK knockdown, and the change was not statistically significant by ANXA7 knockdown in Hca-P cells as evidenced by RNA-sequencing (fold change is 0.741 and 1.081, respectively, of control-shRNA; *P* < 0.05 and *P* > 0.05, respectively). The cis-targeted gene of ENSMUST00000130486 is *CD55*, the expression of *CD55* was significantly reduced by JNK knockdown, and the change was not statistically significant by ANXA7 knockdown in Hca-P cells as evidenced by RNA-sequencing (fold change is 0.359 and 0.544, respectively, of control-shRNA; *P* < 0.01 and *P* > 0.05, respectively). The cis-targeted gene of ENSMUST00000197932 is *Olfr266*, the expression of *Olfr266* was both significantly increased by JNK knockdown and by ANXA7 knockdown in Hca-P cells as evidenced by RNA-sequencing (fold change is 7.721 and 7.125, respectively, of control-shRNA; both *P* < 0.01) (Table 3).

## 4. Discussion

Long noncoding RNAs are involved in multiple cellular processes including gene dosage compensation, epigenetic regulation, the cell cycle, and differentiation [34], and dysregulation of lncRNA pathways modulating these processes underlies several human disorders caused by chromosomal deletions and translocations. In addition, lncRNAs have also been linked to multiple types of cancer, including HCC [35]. For instance, the lncRNAs HOXD-AS1 [36], HOTAIR [37], and MALAT1 [38] have been reported to facilitate HCC tumorigenesis. Therefore, screening for HCC-associated lncRNAs may be a fruitful approach for identifying novel therapeutic targets.

ANXA7 modulates the growth, differentiation, proliferation, secretion, invasion, migration, and apoptosis of various tumor cells [12, 39, 40] and is a key regulator of early-stage hepatic cancer lymphatic metastasis in mice [4]. To the best of our knowledge, however, no study has examined if ANXA7 regulates specific lncRNAs in HCC cells. The MROH7-TTC4 read-through lncRNA is upregulated by inhibition of ANXA7 GTPase activity and in turn suppresses vascular endothelial cell apoptosis [41]. Further, the ANXA7 inhibitor ABO alters the expression and distribution of the lncRNA CERNA1 in vascular endothelial cells [42]. Similarly, we found that ANXA7 knockdown enhanced expression of ENSMUST00000197932 and downregulated the expression of NONMMUT012084.2, NONMMUT024756.2, and ENSMUST00000130486. While the specific contributions of these species to HCC require further study, our bioinformatics analyses of predicted target genes suggested involvement in cellular processes related to carcinogenesis (discussed below).

JNK is a stress-responsive signaling molecule known to induce tumorigenic responses (such as cell proliferation, survival, metastasis, and migration) to carcinogenic stimuli. Li et al. found that the lncRNA BANCR regulates vascular smooth muscle cell (VSMC) proliferation and migration partly by activating the JNK pathway [43], while Zhang et al. reported that suppressing the lncRNA H19 decreased the expression of JNK pathway proteins in human lung cancer cells [44]. Gao et al. found that the lncRNA MALAT-1 blocked JNK signaling, thereby suppressing IL-1*β*-induced inflammation of articular chondrocytes, chondrocyte apoptosis, and extracellular matrix degradation, and enhancing chondrocyte proliferation [45]. A recent study reported that the lncRNA LINC00707 promotes HCC through activation of the ERK/JNK/AKT signaling pathway [46]. Modulation of IGF2BP1 by the lncRNA HCG11 also suppressed apoptosis of HCC cells via MAPK signaling [47]. Similarly, we found that JNK knockdown enhanced expression of ENSMUST00000197932 and downregulated the expression of NONMMUT012084.2, NONMMUT024756.2, and ENSMUST00000130486. Therefore, JNK signaling may include lncRNAs such as those differentially expressed in the current study.

We identified 4 novel lncRNA differentially expressed under JNK knockdown and under ANXA7 knockdown. As none of these species has been previously characterized, we predicted functions based on putative target genes (Table 2). The cis-targeted gene of NONMMUT012084.2 is *Rdm1*, which is involved in DNA double-strand break repair, RNA processing, and protein translation [48]. Loss of *Rdm1* enhanced HCC progression via p53 and Ras/Raf/ERK pathways [49], strongly suggesting that NONMMUT012084.2 indeed functions in HCC. The cis-target gene of NONMMUT024756.2 is *Irak4*, encoding interleukin-1 receptor-associated kinase 4. The IRAKs (subtypes 1–4) are serine-threonine kinases involved in toll-like receptor and interleukin-1 signaling pathways, through which they regulate innate immunity and inflammation [50]. The rs4251545 polymorphisms of IRAK4 (p.Ala428Thr) were reported to modify susceptibility to hepatitis B virus (HBV)-related HCC via increased proliferation and reduced production of inflammatory cytokines and chemokines [51]. Further, IRAK1 augmented HCC cell stemness and drug resistance via AP-1/AKR1B10 signaling [52]. The cis-targeted gene of ENSMUST00000130486 is *Cd55*, a member of the regulators of complement activation (RCA) family known as a decay-accelerating factor (DAF). This DAF appears to exert activities beyond immunological regulation such as promotion of tumorigenesis. However, there are few studies of CD55 in liver cancer. Finally, the cis-targeted gene of ENSMUST00000197932 is *Olfr266*, encoding olfactory receptor 266. Like ENSMUST00000130486 and CD55, the functions of this lncRNA and cis target in HCC require further investigation.

In our study, we found that after JNK was downregulated, the expression of NONNMMUT012084.2, NONNMMUT024756.2, and ENSMUST000000130486 was decreased, and their target genes *Rdm1*, *Irak4*, and *CD55* were also decreased. However, when ANXA7 was downregulated, the expression of NONNMMUT012084.2, NONNMMUT024756.2, and ENSMST00000130486 was decreased, and the expression of target genes *Rdm1*, *Irak4*, and *CD55* was not significantly changed. When JNK and ANXA7 were downregulated, the expression of ENSMUST000000197932 and its target gene *Olfr266* were significantly increased (Table 3). In recent years, through the study of lncRNAs, researchers have found that lncRNA can regulate gene expression at multiple levels. Generally speaking, lncRNAs mainly include the following such levels: some specific lncRNAs recruit chromatin remodeling and modification complexes to specific sites to change DNA/RNA methylation status, chromosome structure, and modification status, and then control the expression of related genes. In eukaryotic cells, transcription factors are very important for gene transcription. They can bind to RNA produced by gene transcription and control RNA transcription, localization, and stability. Some lncRNAs, as ligands, bind with some transcription factors to form complexes and control gene transcription activity. In addition to the above two mechanisms, lncRNAs are also directly involved in the posttranscriptional regulation of mRNA, including variable shear, RNA editing, protein translation, and translocation. These processes are very important for gene functional polymorphism. In addition to direct regulation of mRNA, lncRNAs also affect the expression of target genes by controlling miRNA expression. The regulation of lncRNAs on target genes is complex and diverse, and its specific mechanism needs further experimental exploration. The functions of the four lncRNAs in this study are all new and their complex and diverse effect on target genes and the development of hepatocellular carcinoma need further exploration in the future. This paper is intended to provide new directions for researchers.

In recent years, several lncRNAs have been implicated in human diseases, including malignant tumors [53], and the underlying mechanism is a major focus of current liver cancer research [27–30]. Biological processes with the greatest number of predicted target genes of differentially expressed lncRNAs included “biological regulation,” “cellular process,” “metabolic process,” “regulation of biological process,” “response to stimulus,” and “single-organism process.” Most of these target genes were associated with “membrane and organelle” and participated in “binding,” “catalytic activity,” “molecular function regulator,” “molecular transducer activity,” “signal transducer activity,” “transporter activity,” and “nucleic acid binding transcription factor activity.” The top 30 KEGG pathways of these target genes in shRNA-ANXA7 cells included “ubiquitin mediated proteolysis,” “cell cycle,” and “DNA replication” among other functions. In the shRNA-JNK target gene group, the top 30 KEGG pathways were “ubiquitin-mediated proteolysis,” “Fanconi anemia pathway,” and “ribosome biogenesis in eukaryotes.” Therefore, these differentially expressed lncRNAs appear to modulate a plethora of diverse cellular pathways, and specific associations with cancer-related processes require further study.

In this study, we screened for lncRNAs regulated by ANXA7 and JNK in hepatoma cells by RNA sequencing of stable ANXA7 and JNK knockdown cell lines and conducted bioinformatics analyses to identify species with a high probability of involvement in cancer-related cellular processes. Collectively, these results strongly suggest that both ANXA7 and JNK contribute to liver carcinogenesis and metastasis through the regulation of lncRNAs expression. These findings provide additional clues to ANXA7- and JNK-dependent mechanisms of liver cancer progression and novel molecular targets for therapeutic intervention.

## Figures and Tables

**Table 1:** PCR primer sequences.

Name of gene	Forward primer	Reverse primer
NONMMUT012084.2	5′-CCAGGGTGCTCCCTACATGT-3′	5′-CCAAAAAGCCACTTAAGGTGTCA-3′
NONMMUT024756.2	5′-GACCGGAAAAGCACTTTGACA-3′	5′-CGGTGCCAACTCGTCTATTAACT-3′
ENSMUST00000197932	5′-AAGCTGCTTTGACCCAGCAA-3′	5′-GAGAAGCCGGAGTTTCTGTAGTG-3′
ENSMUST00000130486	5′-GAGCATGCTAGCTCCCACAGT-3′	5′-GGCAGCTTGTCCAATCTGAGA-3′
GAPDH	5′-AGGTCGGTGTGAACGGATTTG-3′	5′-TGTAGACCATGTAGTTGAGGTCA-3′

**Table 2:** Prediction of cis-regulated target genes.

lncRNA_ID	Target gene_ID	Target gene_name
NONMMUT012084.2	ENSMUSG00000010362	Rdm1
NONMMUT024756.2	ENSMUSG00000059883	Irak4
ENSMUST00000130486	ENSMUSG00000026399	CD55
ENSMUST00000197932	ENSMUSG00000043529	Olfr266

**Table 3:** The RNA-sequencing results of cis-regulated target genes.

Target gene	shRNA-JNK vs control-shRNA		shRNA-ANXA7 vs control-shRNA	
	Fold change	*P* value	Fold change	*P* value
Rdm1	0.667	<0.01	4.157	>0.05
Irak4	0.741	<0.05	1.081	>0.05
CD55	0.359	<0.01	0.544	>0.05
Olfr266	7.721	<0.01	7.125	<0.01

## Data Availability

The datasets used and/or analyzed during the current study are available from the corresponding author on reasonable request.
